# Risk factors for stillbirth at term: an Italian area-based, prospective cohort study

**DOI:** 10.1016/j.xagr.2023.100269

**Published:** 2023-09-18

**Authors:** Cristina Salerno, Beatrice Melis, Valeria Donno, Gloria Guariglia, Daniela Menichini, Enrica Perrone, Fabio Facchinetti, Francesca Monari

**Affiliations:** 1 Obstetrics and Gynecology Unit, Mother-Infant and Adult Department of Medical and Surgical Sciences, University of Modena and Reggio Emilia, Modena, Italy (Drs Salerno, Melis, Donno, Guariglia, Facchinetti, and Monari); 2 International Doctorate School in Clinical and Experimental Medicine, Department of Biomedical, Metabolic and Neural Sciences, University of Modena and Reggio Emilia, Modena, Italy (Dr Menichini); 3 Emilia-Romagna Health and Welfare Directorate, Community Care Department, Bologna, Italy (Dr Perrone)

**Keywords:** access to antenatal cares, fertility treatments, prevention, risk factors, small for gestational age, stillbirth, term pregnancy

## Abstract

**BACKGROUND:**

Stillbirth at term has great emotional impact on both parents and professionals. In developed countries, efforts to identify risk factors are mandatory to plan area-specific prevention strategies.

**OBJECTIVE:**

The aim of the study was to identify independent risk factors that contribute to stillbirth at 37 weeks’ gestation or later.

**STUDY DESIGN:**

This was an area-based, prospective cohort study on pregnancy at term with enrolled from 2014 to 2021 in Emilia-Romagna, a north Italian region. Data were retrieved from both birth certificates and the Stillbirth Surveillance system database. To identify independent risk factors, a multivariate analysis using logistic regression was performed. A descriptive analysis of the causes of stillbirth is also reported.

**RESULTS:**

In the observation period, 246,437 babies born at term (including 260 stillbirths, giving a rate of 1.06/1000) were considered. The risk factors independently associated with stillbirth were small for gestational age babies (odds ratio, 2.58; 95% confidence interval, 1.88–3.53), pregnancy achieved though fertility treatments (odds ratio, 2.01; 95% confidence interval, 1.15–3.51), and delayed access to pregnancy services (odds ratio, 1.56; 95% confidence interval, 1.10–2.22). In multipara, the presence of a previous stillbirth (odds ratio, 3.91; 95% confidence interval, 1.98–7.72) was also associated with an increased risk for recurrence. Early- rather than late-term was an additional risk factor. The most frequent causes of death were placental and cord disorders (61/260 and 56/260, respectively). However, 28.1% of cases remain unexplained.

**CONCLUSION:**

The risks for stillbirth at term are known early in pregnancy or could be identified through tailored antenatal management, allowing effective preventive strategies to reduce preventable cases.

**AJOG Global Reports at a Glance**

**Why was this study conducted?**

Stillbirth at term is poorly analyzed despite its impact on parents and healthcare professionals. Moreover, it is mandatory to recognize local determinants when designing specific interventions.

**Key findings**

Independent risk factors for stillbirth at term were early- rather than late-term gestation, pregnancy following fertility treatments, small for gestational age baby, delayed access to pregnancy services, and the presence of a previous fetal death in multipara.

**What does this add to what is known?**

In certain settings, late-term pregnancy does not represent a risk factor, whereas fetal growth anomalies and obstetrical history confirm previous data. Many risk factors are present early in pregnancy, thus allowing incorporation of preventive strategies.

## Introduction

Stillbirth (SB) is deemed by the global health community to be a marker of the health system's quality of care during pregnancy and childbirth. Although significant reductions in SB rates in high-income countries have been achieved in the last decades, rates have stabilized or decreased marginally in many regions.^1^^,^^2^

The risk for SB at term varies from 1.1 to 3.2 per 1000 pregnancies in developed countries. SB ≥37 weeks’ gestation is less studied than the entire phenomenon. Muglu et al^3^ found that the overall gestation week–specific prospective risk for SB gradually increased with gestational age from 0.11 per 1000 pregnancies at 37 weeks’ gestation to 3.18 per 1000 pregnancies at 42 weeks’ gestation. In the literature, there are a few studies that evaluated SB at term overall; most of the research analyzed risk factors individually or partly, which sometimes have mixed results.^4^, ^5^, ^6^, ^7^ To the best of our knowledge, there are only 2 systematic reviews and meta-analyses on the risk factors for fetal death at term. The main ones included advanced maternal age, low maternal education level, smoking habits, prepregnancy overweight and obesity, multiparity, diabetes, hypertension, and fetal growth restriction.^8^^,^^9^

Research on SB at term is mandatory to improve the prevention strategies to minimize this adverse perinatal outcome. The aim of our study was to identify independent risk factors that contribute to SB at 37 weeks’ gestation or later.

## Materials and Methods

This was an area-based, prospective cohort study that was undertaken from January 2014 to December 2021. All women who gave birth at term (≥37 weeks of gestation) in every birth center of the Emilia-Romagna region, northern Italy, were included in this study. The only exclusion criterion was pregnancies ending prematurely. Gestational age was estimated based on the last menstrual period or on the first ultrasound examination if the last menstrual period was unknown or unreliable.

Maternal and neonatal information and details regarding pregnancy and delivery were collected from birth certificates of the Emilia-Romagna region, which have been published and analyzed yearly since 2002.^10^ This is the official digital anonymous repository that collects reliable and accurate information about all births and about the parents’ sociodemographic data in the interest of public health.

Prepregnancy body mass index (BMI) ≥25 kg/m^2^ was defined as overweight. The gestational weight gain and the neonatal anthropometry were evaluated using the Institute of Medicine recommendation and the Italian Neonatal Study charts, respectively.^11^^,^^12^ Small for gestational age (SGA), adequate for gestational age (AGA), or large for gestational age (LGA) were defined if the neonatal birthweight percentile was <10%, between 10% and 90%, or >90%, respectively. We considered delayed access to pregnancy care service to be when the first pregnancy visit was made at 13 weeks’ gestation or later. At a minimum, 4 antenatal visits were considered appropriate for optimal surveillance.^13^

Moreover, in the Emilia-Romagna region, there is a Surveillance System of Perinatal Mortality that has been active since 2014.^14^ A specific SB clinical diary and the diagnostic work-up was uniformly applied in the area. Six local audit groups and a central audit group, including at least an obstetrician, a neonatologist, and a pathologist, collected and discussed every case of SB that occurred in the region. The causes of death were established according to Re.Co.De. classification.^15^ To ensure precision, SB identified through birth certificates were compared with those identified from the surveillance system, reaching 100% identification of the cases and a 5% disagreement between the 2 systems.

Data were analyzed using statistical package Stata 16.1 (StataCorp, College Station, TX). Continuous variables are reported as mean±standard deviation (SD). Categorical variables are reported as the absolute and percentage frequencies. A *P* value <.05 was considered statistically significant. The comparisons between the SB and the alive newborns groups were made using the student *t* test for continuous variables and the chi-square test or the Fisher exact test, when appropriate, for categorical variables. Multivariate analyses were performed to investigate the risk factors associated with an increased risk for SB. Results of logistic regression are reported as the odds ratio (OR) with 95% confidence interval (95% CI) and the *P* value. A descriptive analysis on the distribution of causes of death was performed.

The Ethical approval for this study was obtained from the local institutional review board (35265 – November 24, 2021). Information was stored anonymously in a secure database. Informed consent for the diagnostic work-up was not required, because diagnostic investigations are mandatory by law in the case of SB in Italy (D.M. 7/2014 and D.P.C. 170/99). Women and newborn privacy were ensured during all the phases of data collection and analysis.

## Results

**Figure 1:**
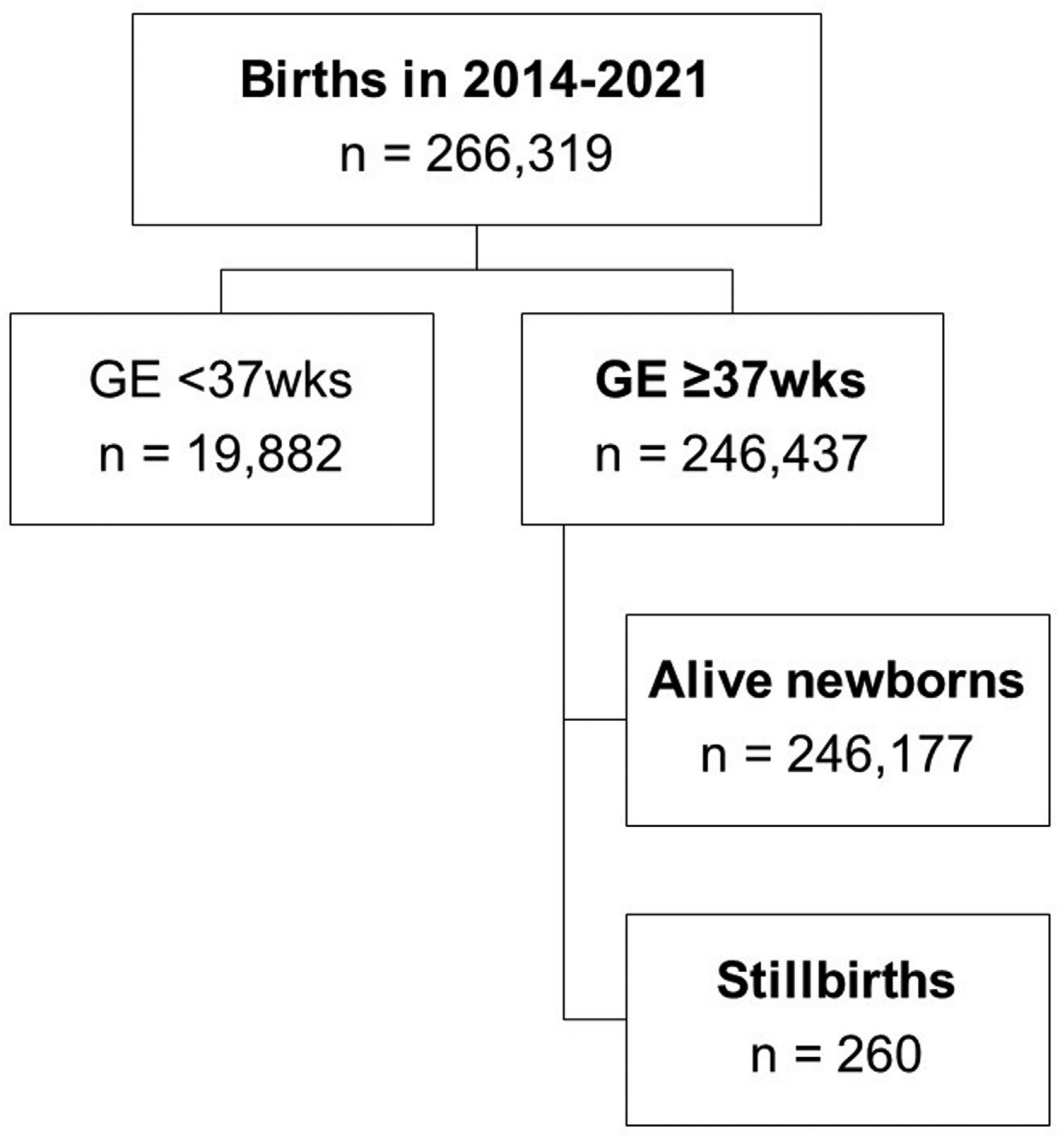
Flow chart of study population *GE*, gestational age.

There were 246,437 births at ≥37 weeks’ gestation of 266,319 births that occurred from January 2014 to December 2021. These included 260 fetal deaths, giving an overall SB rate at term of 1.06 per 1000 births (Figure 1).

Salerno. Risk factors for stillbirth at term. Am J Obstet Gynecol Glob Rep 2023.

Risk factors for SB identified in the univariate analysis are reported in Table 1. The risk at >41 weeks’ gestation is lower than that at 39 to 40 weeks’ gestation (OR, 0.55; *P*=.024). However, the impact of gestational age has been analyzed thoroughly with stratification of the risk by gestational week within the cohort of ongoing pregnant women. The ratio of SB remained stable in the range of 39 to >41 weeks’ gestation (Table 2).

**Table 1:** Univariate analysis of risk factors for stillbirth at term

Risk factors	Stillbirths n=260	Alive newborns n=246,177	*P* value
Gestational age 37–38 wk	111 (42.7)^a^	62,529 (25.4)	<.001
Gestational age 39–40 wk	128 (49.2)	141,306 (57.4)	Ref
Gestational age ≥41 wk	21 (8.1)	42,342 (17.2)	.024
Median maternal age (y)	32.4±5.7	32.1±5.5	.298
Maternal age ≥40 y	32 (12.3)	21,060 (8.6)	.055
Low maternal education (<8 y)	68 (26.2)	60,151 (24.4)	.428
Foreign maternal origin	107 (41.2)	88,801 (36.1)	.097
Smoking habit	32 (12.3)	36,501 (14.8)	.205
Multiparity	136 (52.3)	122,832 (49.9)	.437
Previous stillbirth	7 (5.1)	1822 (1.5)	.001
Interpregnancy interval (y)	4.4±2.9	4.7±3.2	.162
Prepregnancy BMI ≥25 kg/m^2^	90 (34.6)	69,462 (28.2)	.023
Excessive gestational weight gain	61 (23.5)	49,089 (19.9)	.186
Multiple pregnancy	5 (1.9)	3633 (1.5)	.151
Assisted reproduction	13 (5.0)	6263 (2.5)	.014
SGA newborn	50 (19.2)	21,105 (8.6)	<.001
LGA newborn	15 (5.8)	25,567 (10.4)	.065
Female gender	137 (52.7)	119,817 (48.7)	.093
Delayed access to pregnancy cares	39 (15.0)	24,374 (9.9)	.004
Prenatal visit <4	13 (5.0)	7664 (3.1)	.071

*BMI*, body mass index; *LGA*, large for gestational age; *SGA*, small for gestational age.

a Data are presented as number (percentage).

**Table 2:** Risk for stillbirth calculated in the cohort of ongoing pregnancies, stratified by gestational age

Gestational age (wk)	Stillbirths	Alive newborns	Cohort	Rate (per 1000 births)
37	55	17,332	246,437	0.223
38	56	45,197	229,050	0.244
39	74	72,013	183,797	0.403
40	54	69,293	111,710	0.483
≥41	21	42,342	42,363	0.496
Total	260	246,177		

A significant correlation was observed between SB and pregnancy achieved through fertility treatment (OR, 1.99; *P*=.014), SGA newborn (OR, 2.47; *P*<.001), prepregnancy overweight (OR, 1.35; *P*=.023), and delayed access to pregnancy care service (OR, 1.63; *P*=.004). Moreover, women who already experienced a SB (OR, 3.51; *P*=.001) also had an increased risk for SB (Table 1).

In the multivariate analysis (Table 3), the risk for SB was statistically significant for SGA newborns (*P*<.001), fertility treatment (*P*=.015), and delayed access to pregnancy services (*P*=.013); in multipara, the presence of a previous SB was also associated with an increased risk for fetal death (*P*<.001).

**Table 3:** Multivariate analysis of risk factors for stillbirth at term

Risk factors	OR	95% CI	*P* value
Pre-pregnancy BMI ≥25 Kg/m^2^	1.25	0.96–1.63	.092
Assisted reproduction	2.01	1.15–3.51	.015
SGA newborn	2.58	1.88–3.53	<.001
Delayed access to pregnancy cares	1.56	1.10–2.22	.013

Risk factors (multiparous women only)	OR	95% CI	*P* value
Previous stillbirth	3.91	1.98–7.72	<.001
SGA newborn	2.60	1.68–4.01	<.001
Delayed access to pregnancy cares	1.64	1.05–2.57	.030

*BMI*, body mass index; *CI*, confidence interval; *OR*, odds ratio; *SGA*, small for gestational age.

**Figure 2:**
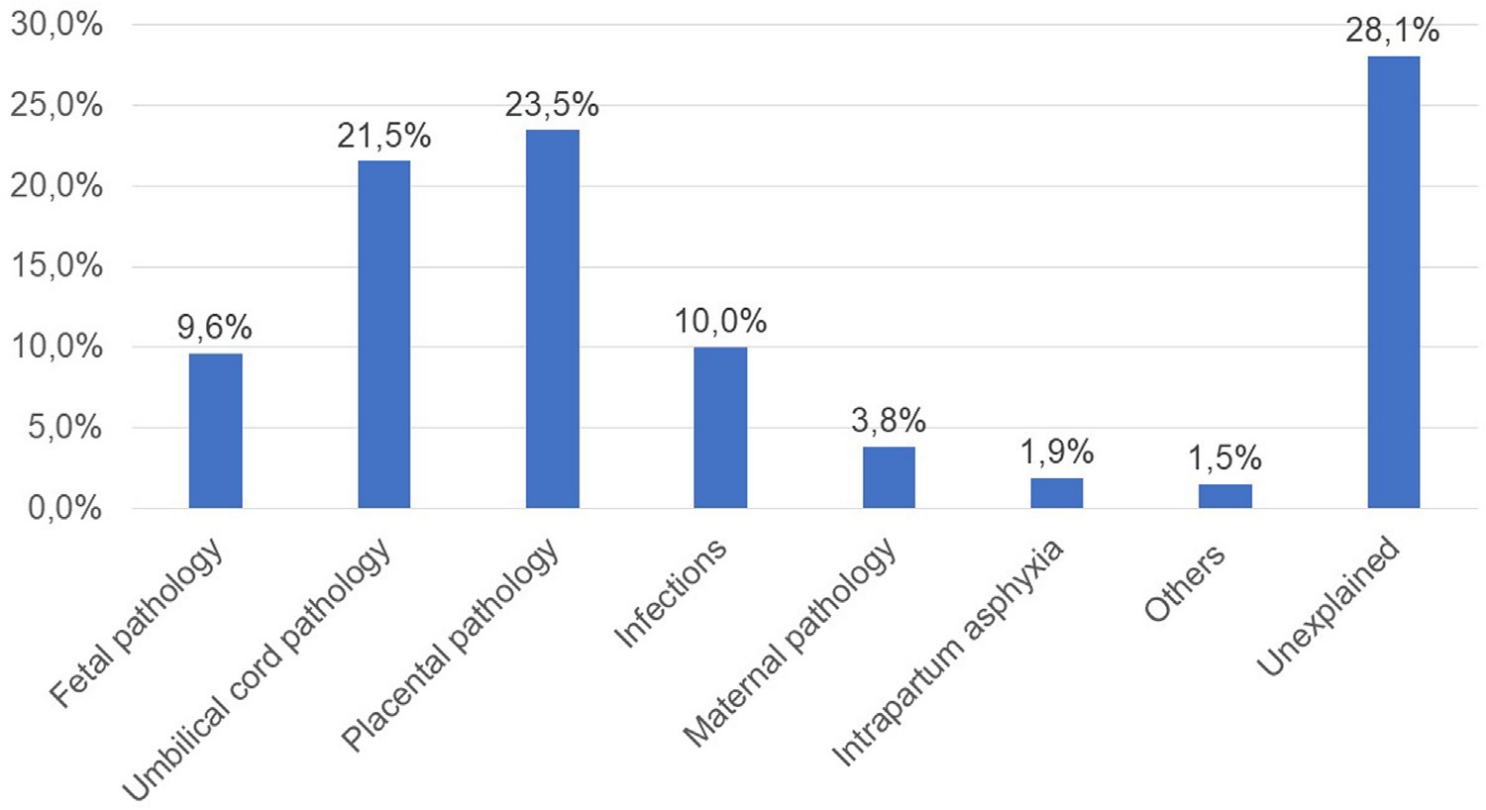
Causes of death according to the modified Re.Co.De. classification

According to modified Re.Co.De. classification, the most frequent causes of SB at term (Figure 2) were placental and umbilical cord pathology (61/260 and 56/260, respectively). In addition, there were 26 cases of infections (10.0%) and 25 cases of fetal disorders (9.6%). However, 73 cases (28.1%) remained unexplained despite performing complete diagnostic tests for 56 of them (21.5%). As shown in Table 4, few differences exist in the causes of death by gestational age groups. This also includes unexplained SB.

Salerno. Risk factors for stillbirth at term. Am J Obstet Gynecol Glob Rep 2023.

**Table 4:** Causes of death in relation to the gestational age

Causes of death	37–38 weeks n=111	39–40 weeks n=128	≥41 weeks n=21	*P* value
Fetal disorders	12 (10.8)^a^	10 (7.8)	3 (14.3)	.85
Umbilical cord disorders	25 (22.5)	27 (21.1)	4 (19)	.76
Placental disorders	32 (28.8)	25 (19.5)	4 (19)	.22
Infections	8 (7.2)	15 (11.7)	3 (14.3)	.62
Maternal disorders	4 (3.6)	6 (4.7)	0	.53
Intrapartum asphyxia	0	4 (3.1)	1 (4.8)	.15
Unexplained stillbirth	30 (27)	41 (32)	6 (28.6)	.39

a Data are presented as number (percentage).

## Discussion

### Principal findings

Our study demonstrated that an SGA infant, a pregnancy following fertility treatment, delayed access to pregnancy services, and, in multiparous women, the presence of a previous fetal death are independent risk factors for SB at term. Gestation up to 37 to 38 weeks’ gestation also has an increased risk for SB, whereas advanced gestational age (41 weeks or later) did not represent a risk factor in our population.

## Results

To the best of our knowledge, there are only 2 similar studies in the literature. The largest one, a Korean retrospective cohort study in singleton term pregnancy, demonstrated that the risk for SB was significantly higher with the increasing maternal age, in the case of SGA and LGA newborns, nulliparity, lower maternal education level, and foreign maternal nationality.^16^ In another retrospective cohort study, the main risk factors were suspected intrauterine growth restriction, diabetes, hypertensive disorders, advanced maternal age, and grandmultiparity.^17^ Such heterogeneous findings may be related to the unique features of the study populations and the characteristics of pregnancy care services. The latter study also found that the advanced gestational age was not significantly associated with antepartum fetal death (*P*<.001), confirming our actual data.^17^ Moreover, a recent, single-center, retrospective cohort study conducted on more than 50,000 term deliveries reported the highest incidence of SB at 38 weeks of gestation.^18^ Overall, the above findings are consistent with our results, but they contradict previous studies that found that SB was more common among late-term and post-term pregnancies.^19^^,^^20^ Possible explanations include the following: first, advanced gestational age has long been recognized as a risk factor for SB, so in recent years, late-term and post-term pregnancies were followed more closely; second, pregnancies are interrupted earlier because of the presence of complications (eg, preeclampsia, intrahepatic cholestasis, diabetes mellitus, SGA), thus allowing only uncomplicated pregnancies to reach the late- and post-term periods.

Several other studies evaluated only a single risk factor, namely SGA newborns.^21^^,^^22^ Data from different cohorts, including the cohort of this study, universally found that impaired fetal growth is definitely a risk factor for SB at term, a risk that increases further with advancing gestation and that is highest among babies with a weight below the third percentile.^23^ Placental disorders play a major role and, as reported in our series, SB is typically a consequence of placental maternal vascular malperfusion.^24^^,^^25^

In this study, we confirmed the findings of previous studies on the risk of pregnancy achieved following fertility treatments.^26^^,^^27^ However, it is not clear if the increased risk is related to the fertility treatment or to the underlying subfertility causes.^28^ Anyway, placental examination gave a rationale for such an increased risk,^29^ in addition to the known complications of in vitro fertilization such as preeclampsia, placental abruption, or SGA newborns.^30^^,^^31^

Contrary to previous studies, we did not find any correlation between term SB and multiple pregnancies despite the increased risk related to fertility treatments that often lead to this kind of gestations.^32^^,^^33^ The higher probability of preterm delivery and the actual trend to reduce multiples in fertility treatments may account for such differences.

As far as recurrence is concerned, some studies agree with our results, but none of them specifically addressed term pregnancy.^34^^,^^35^ A recent Canadian retrospective cohort study demonstrated that the overall risk for recurrent antepartum SB is low, but substantially increased in the case of previous SGA-related SB.^36^

Increased prepregnancy BMI is also well recognized as a risk for SB. Overweight and obesity are characterized by several adverse outcomes, including preeclampsia, SGA newborns, fetal growth restriction, and gestational diabetes.^37^, ^38^, ^39^ Our findings were unable to capture this association, possibly because of the over-representation of other risk factors identified through antenatal surveillance that was seldom included in other studies.^16^, ^17^, ^18^ Indeed, our data suggest that there is a significant association between delayed access to pregnancy services and term SB, confirming some previous findings.^40^^,^^41^ Insufficient prenatal care is associated with an increased SB rate irrespective of the presence of high-risk conditions, suggesting a possible causal relationship.^42^

### Clinical implications

These findings underline the relevance of risk factors that are either known from the beginning of pregnancy and/or could be susceptible to modification. Overall, antenatal surveillance (fetal growth) and the right timing of delivery seem to be focused strategies.^21^^,^^36^ It is of utmost importance to stimulate early access to pregnancy care services (within the first trimester) for an adequate implementation of therapeutic or prophylactic actions. The early detection of even unmodifiable risk factors (pregnancy following fertility treatment or the presence of a previous SB) empowers antenatal management despite the uncertain efficacy of fetal movements monitoring.^43^^,^^44^ Finally, the higher risk for SB at early-term gestation underlines the relevance of third-trimester management and perhaps the implementation of fetal growth assessment at 35 to 37 weeks’ gestation. In addition, careful monitoring of advanced gestational age typical of our setting can detect pregnancy complications, which can lead to a significant reduction in late-term SB.

### Research implications

Future research is needed to confirm our data. Most importantly, this information should be collected in specific areas. Although some risks have been assessed globally (growth restriction, maternal overweight, recurrence), others (late-term) would act only locally because of the relation with antenatal care organization. Indeed, sociocultural conditions, in particular factors involved in the delayed access to pregnancy care services or in their inadequate use, could only be studied in a context-specific situation.

### Strengths and limitations

The strengths of our study include (1) the prospective design in a defined period; (2) the data collection for SB that was enabled by a regional surveillance system that also provided evaluation of the causes of death, and (3) the inclusion of a relatively large cohort of women.

This study has some limitations. The sample size was limited, especially in terms of the low SB rate, because it was an area-based collection. In addition, some information was lacking, such as the preexisting maternal conditions or the complications in previous pregnancies. Although we do not know the specific fertility treatments applied in the cohort, it is worth noting that according to the national registry, >90% are represented by in vitro fertilization and intracytoplasmic sperm injection procedures, which are the procedures associated with placenta-related complications.^45^

## Conclusion

SB, specifically those that occur at term, are a heavy burden on the health system, in addition to being a dramatic event for both parents and professionals. Although various studies have been inconclusive in the identification of risk factors, these data suggest that in a high-income country, both clinical (fertility treatments, SGA) and organizational (access to care) burdens are susceptible to improvements. Stimulating earlier access to pregnancy care services and the identification of abnormal fetal growth trajectory in the third trimester are immediate candidate interventions to reduce SB risk at term.
